# Ocular genetics in the Japanese population

**DOI:** 10.1007/s10384-024-01109-8

**Published:** 2024-09-14

**Authors:** Yoshihiro Hotta, Kaoruko Torii, Masakazu Takayama

**Affiliations:** Hamamatsu University School of Medicine, 1-20-1 Handayama, Chuo-ku, Hamamatsu city, Shizuoka, 431-3192 Japan

**Keywords:** Hereditary eye disease, Inherited retinal dystrophy, Retinitis pigmentosa, Early-onset severe retinal dystrophy (EOSRD), Genetic counseling

## Abstract

In today’s globalized society, ophthalmologists can examine people of different ethnicities regardless of where they live. The frequency of disease-causing genes varies according to a patient’s ethnic background. We explain genetic findings for Japanese patients with inherited eye diseases. Ocular genetics has made great advances over the past 30 years. For example, detecting mutations at nucleotide position 11778 in mitochondrial DNA was useful in the genetic diagnosis of Leber’s hereditary optic neuropathy (LHON). I evaluated the genotype-phenotype relationship in cases of corneal dystrophy and inherited retinal dystrophy (IRD). I identified the entire exon sequence of the *eyes shut homolog* (*EYS*) gene in patients with autosomal recessive retinitis pigmentosa (RP). *EYS* gene mutations are the most frequent cause of autosomal recessive RP. *RPGRIP1* may be a common causative gene with early-onset severe retinal dystrophy, including Leber congenital amaurosis. However, some genes have complex structures that are difficult to analyze, including the *OPN1LW/OPN1MW* gene cluster in blue cone monochromacy and the *IKBKG/NEMO* genes in incontinentia pigmenti. This review will also present two cases with uniparental disomy, a case of IRD with double mutations, and a case with RP complicated with LHON-like neuropathy. Precise understanding of the effects of genetic variants may reveal differences in the clinical characteristics of patients with the same variant. When starting genome medicine, accurately diagnosing the patient, making accurate prediction, determining the genetic pattern, and providing genetic counseling are important. Above all, that both the doctors and patients understand genetic diseases correctly is important.

## Introduction

As people travel more easily and the world becomes more globalized, ophthalmologists can see patients of different ethnicities regardless of location. Inherited eye diseases (IRDs), of which retinitis pigmentosa (RP) is typical, have poor prognoses. Few curative treatments are available other than treatment for complications such as cataracts. Therefore, accurately diagnosing the patient, making the most accurate prediction, determining the inheritance pattern, and providing genetic counseling are important. The extent and significance of genetic contributions to eye disease depend on the disease. RP and color vision defects are caused primarily by genetic abnormalities; age-related macular degeneration and moderate myopia are caused by several genetic predispositions as well as other factors.

Molecular genetics has progressed remarkably in the last three decades. DNA was once amplified using E. coli, but DNA is now amplified using the polymerase chain reaction (PCR). DNA sequencing, which involves analyzing and determining nucleotide fragments, has advanced significantly since the time DNA fragments had to be cloned before being sequenced. The sequence of the entire human genome was roughly determined in AD 2000, so the rapid progress is surprising. The invention of next-generation sequencing allows genome decoding to be outsourced; current databases contain many genome sequences. Here, we review the status of genetic findings to assist the genetic counseling for Japanese patients with inherited eye diseases. Some dates may be out of order, depending on the structure of the content.

## Leber’s hereditary optic neuropathy (LHON)

In 1988, a group at Emory University reported the 11778 mitochondrial gene mutation in LHON (OMIM 535000) [1]. In 1989, a follow-up study was reported from Japan [2]. In the same year, we reported for the first time that genetic analysis using PCR and the restriction enzyme *SfaNI* was useful for diagnosis [3]. Although the prognosis of LHON caused by the 11778 mutation is poor, we experienced a case in which the visual acuity in one eye was restored [4].

Deciphering the high complication rate of the 11778 mutation in patients with LHON was a breakthrough; consequently, mitochondrial gene analyses of patients with LHON in Japan have been performed [5]. To clarify the status of patients in Japan, we conducted a multicenter survey using a questionnaire. We obtained responses from 64 (74.4%) of 86 universities with medical schools in Japan. Although the visual prognosis of LHON complicated by the 11778 mutation is poor, the visual prognosis of this cohort is slightly better than that reported for the USA [6, 7].

## Corneal dystrophy

**Fig 1.**
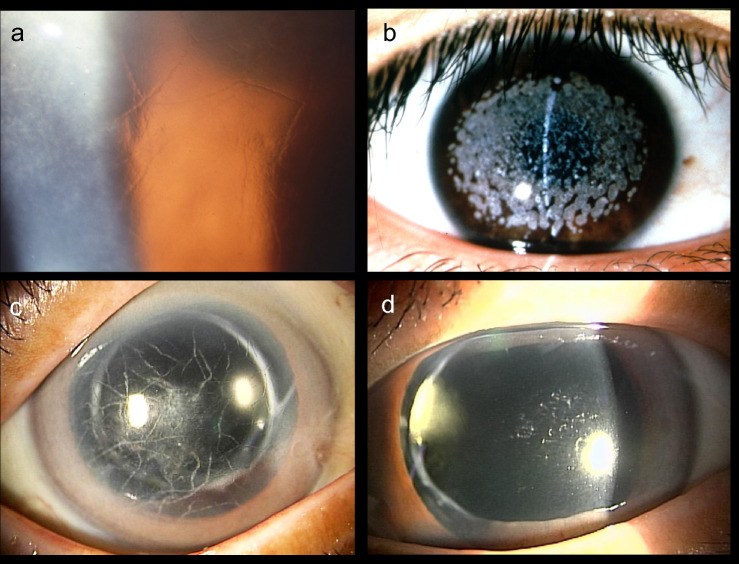
Corneal dystrophy caused by *TGFBI* gene mutations. **a** Lattice corneal dystrophy due to heterozygous R124C mutation (age 57) [21]. A thin grid-like opacity is observed. **b** Avellino corneal dystrophy due to homozygous R124H mutations (age 14) [10]. **c** Right eye and **d** left eye of an 82-year-old patient with lattice corneal dystrophy due to a heterozygous L527R mutation [19]

In 1997, a group at the University of Lausanne reported *TGFBI* gene abnormalities in corneal dystrophy (OMIM 601692) [8]. Following that report, we investigated corneal dystrophy cases at Juntendo University and Nagoya University by the Sanger method. We found that variants of the *TGFBI* gene caused many corneal dystrophies, and the genotype correlated strongly with the phenotype [9–20]. Corneal dystrophies caused by the heterozygous R124H variant often do not cause severe visual dysfunction in adults, but homozygous cases cause visual impairment problems from childhood (Fig. 1) [10]. Lattice corneal dystrophy caused by the heterozygous L527R of the *TGFBI* gene is characterized by thick lattice changes and left–right differences [11, 20, 21]. Although the heterozygous variant of L527R is not clinically problematic until an advanced age, Figure 1 shows an older patient whose eye exhibited intense corneal findings with repeated corneal erosions that caused visual impairment.

## Fundus albipunctatus

In 1999, a group at the Massachusetts Eye and Ear Infirmary reported *RDH5* gene abnormalities in fundus albipunctatus (OMIM #136880) [22]. In 2000, we examined the *RDH5* gene in 14 patients (12 families) with fundus albipunctatus (OMIM 136880) at Nagoya University. Six patients (six families) had concomitant cone dystrophy. All patients had *RDH5* mutations; those with cone dystrophy were older (48–74 years old). Therefore, we speculated that fundus albipunctatus is complicated by cone dysfunction in older age [23]. We also identified a high-frequency Japanese mutation, c.928delinsGAAG (p.L310delinsEV) (Table 1). We also examined the *RDH5* and *RS1* genes in three patients (three families) diagnosed with juvenile retinoschisis (OMIM 312700). The diagnosis was based on several findings, such as electroretinography (ERG), but additional complications occurred due to fundus albipunctatus-like findings. All the patients were diagnosed during childhood, showing pseudo–macular edema and spoke-like appearance in both eyes. The *RDH5* gene mutation was not detected in the three patients; they all had hemizygous mutations in the *RS1* gene [24]. Therefore, we consider that the albipunctatus-like findings in these patients indicate juvenile retinoschisis. The albipunctatus-like findings are also associated with *RLBP1* and *RPE65* variants [25–29].

**Table 1.** Variants frequently detected in Japanese patients with hereditary eye diseases

Gene	Variant		Allele Frequency			
	Nucleotide change	Protein change	ToMMo 54KJPN	gnomAD v4.1.0		
				East Asian	European	Global
*EYS*(NM_001142800.2)	c.4957dup	p.S1653Kfs*2	0.004001	0.001981	0	0.000052
	c.8805C>A	p.Y2935*	0.001974	0.001588	0	0.000043
	c.6557G>A	p.G2186E	0.000627	0.000543	0.000001	0.000015
	c.2528G>A	p.G843E	0.018548	0.009317	0	0.000247
*PDE6B*(NM_000283.4)	c.1604T>A	p.I535N	0.000893	0.000223	0	0.000006
	c.1669C>T	p.H557Y	0.000322	0.000509	0	0.000014
*TACSTD2*(NM_002353.3)	c.352C>T	p.Q118*	0.000700	0.000157	0	0.000004
*RDH5*(NM_002905.5)	c.928delinsGAAG	p.L310delinsEV	NA	NA	NA	NA
*USH2A*(NM_206933.4)	c.2802T>G	p.C934W	0.002155	0.002564	0.000004	0.000090
	c.8254G>A	p.G2752R	0.000332	0.000334	0.000006	0.000015
	c.8559-2A>G	p.?	0.000111	0.000335	0	0.000010
	c.10859T>C	p.I3620T	0.000773	0.000490	0.000001	0.000015

54KJPN: Allele frequency data of SNV/INDEL from short-read whole genome sequencing of 54000 Japanese individuals (https://jmorp.megabank.tohoku.ac.jp/)

gnomAD v4.1.0 (https://gnomad.broadinstitute.org/)

*NA* not applicable

## Retinitis pigmentosa (RP)

RP is the most serious ocular disease, with a frequency of 1 in 3000 to 5000, making it one of the most frequent genetic diseases [30]. Unlike glaucoma, RP has few effective treatments. The clinical presentation is diverse, ranging from patients who are nearly blind at age 20 to those who retain central vision even in old age. This issue is a serious problem in ophthalmology in our aging society. In 1990, the same Massachusetts Eye and Ear Infirmary group reported a mutation in the RP rhodopsin gene [31]. We examined codon 23 and codon 347 of the rhodopsin gene in 19 families with RP considered to have a dominant inheritance pattern at Tohoku University and Juntendo University. A missense mutation of the rhodopsin gene, P347L, was found in one family from Tohoku University. This case was the first report of a variant in RP in Japan [32]. We also identified two families of sectorial RP caused by variants N15S and T17M of the rhodopsin gene [33, 34]. A recent study using a genetic test panel reports that the prevalence of rhodopsin variants in Japanese patients with RP was approximately 2%; R347L was the most commonly detected [35].

The *eyes shut homolog* (*EYS*) gene is one of the major genes associated with autosomal recessive RP [36–38]. We extracted genomic DNA from the peripheral blood of 100 patients with RP, excluding cases with obvious autosomal dominant inheritance, analyzing exons 1–44 of the *EYS* gene by PCR direct sequencing. The *EYS* gene is a large (2 Mb) gene important for the morphogenesis of the *Drosophila* compound eye. We identified seven variants of the *EYS* gene in 18 of the 100 patients. We identified a frameshift mutation (c.4957dupA, due to an insertion) in 12 of the 18 patients and a nonsense mutation (c.8805C>A, p.Y2935X) in 4 of the patients. These two variants are the most frequent gene mutations in Japanese patients with RP. These results were reported by two institutions simultaneously [39, 40]. We focused on the two prominent variants and screened 32 Korean patients with autosomal recessive RP. All 32 Korean patients were from Gyeongsangbuk-do. We identified frameshift mutations due to insertions in two of the 32 patients and C-to-A nonsense mutations in one of them. We screened 19 Japanese patients with autosomal dominant and 28 with Leber congenital amaurosis (LCA) for the two mutations above, but the two mutations were not found in this cohort. Increasing utilization of next-generation sequencing technology in genetic analysis has enabled extensive studies on different retinal dystrophies in Japan [41–43]. Large-scale analysis by whole-exome sequencing (WES) showed that the *EYS* gene aberrations are predominant in retinal dystrophies in Japan, including RP (Table 1) [44]. When Japanese parents who are both suffering from RP consider having children, the probability that the offspring will be homozygous or compound heterozygous for the *EYS* variant cannot be ignored.

When gene variants are common, determining whether the variant is the cause of a disease is challenging; this is known as the variant of unknown significance. The *EYS* gene variant G843E has a very high allelic frequency as a possible cause of diseases showing Mendelian inheritance. In 2012, we could not determine whether this allele is pathogenic because of its extremely high allelic frequency and the lack of homozygous patients in our cohort; however, *EYS* variants in RP have been reported, including this variant [45–48]. This allele is also reported to cause *EYS* dysfunction, mildly affecting the phenotype. It may coexist in a patient heterozygous for the c.5797C>T, p(R1933X) variant of the *RP1* gene and G843E *EYS* allele. Both reports propose a novel non-Mendelian inheritance [49, 50]. We anticipate further progress in research in this direction and advances in gene analysis techniques, including long-range PCR, functional analysis to confirm the effects of gene mutations, and gene editing [48, 51].

Usher syndrome is caused by a *USH2A* genetic abnormality (OMIM 608400) and will be discussed later. RP caused by variants of *USH2A* (nonsyndromic) is common in Europe and the United States of America (USA); a detailed clinical description has been reported [52, 53]. Among the RP cohort described in the previous paragraphs, we analyzed 82 cases by PCR direct sequencing of exons 1–73 of the *USH2A* gene, excluding 18 cases with an *EYS* mutation. In 3 of the 82 cases, a variant was identified as the possible cause of the disease; the variant differs from the spectrum of mutations in Europe and the USA [54]. Among these patients, one with RP15H exhibited homozygosity for the *USH2A* gene variant highly prevalent in Japan and was diagnosed with Usher syndrome based on abnormal hearing test results. Despite a follow-up period of >10 years for the patient with RP15H, hearing loss was identified only after a genetic analysis. In our initial cohort, we frequently observed that individuals retain their vision until they reach 30 years of age. However, we cannot assert this finding definitively because of the low number of patients. In addition, visual field narrowing is considerably advanced in patients in their 30 s [55]. As the number of cases increases, genetic diagnoses are anticipated to prove valuable to clinicians for determining prognoses.

Next-generation sequencers can identify the causative genes in 30–40% of patients with RP [41–43]; however, the challenge lies in the failure to detect these genes in the remaining patients. *EYS*, *USH2A*, and *RP1* (which also cause autosomal dominant RP) are the most common causative genes for autosomal recessive RP. Rhodopsin and *PRPF31* are causative genes for autosomal dominant RP. *RPGR* causes X-linked RP (XLRP). Once the causative gene is determined, predicting the inheritance pattern is often possible, even in sporadic cases. However, genetic penetrance and complicated inheritance, such as pseudo-dominant inheritance and isodisomy, present challenges.

## Early onset severe retinal dystrophy (EOSRD)

Pediatric IRDs includes many diseases ranging from relatively mild to severe visual impairment. They also include cases with one of the symptoms of a systemic syndrome. Pediatric IRDs are less common than RP. LCA (MIM 204000) is rare and the most serious IRD. LCA is a genetically and clinically heterogeneous disease. The diagnosis of LCA is sometimes confusing and diagnosed as early-onset severe retinal dystrophy (EOSRD). We previously conducted a target sequencing study of 34 families with LCA at the National Center for Child Health and Development (NCCHD), Hamamatsu University School of Medicine, The Jikei University School of Medicine, and the University of Occupational and Environmental Health. However, this cohort is not consecutive and has a bias in specimen collection. All patients met the following criteria: (1) severe visual impairment (e.g., nyctalopia, nystagmus, very poor or absent ocular pursuit, or oculodigital sign) within the first year after birth; (2) a severely reduced or non-detectable ERG; and (3) no systemic abnormality other than neurodevelopmental delay at the time of examination. Most of the causative genes accounted for only a single pedigree, except *CRB1* (three families), *NMNAT1* (three families), *RPGRIP1* (three families), and *GUCY2D* (two families) [56–58].

**Fig 2.**
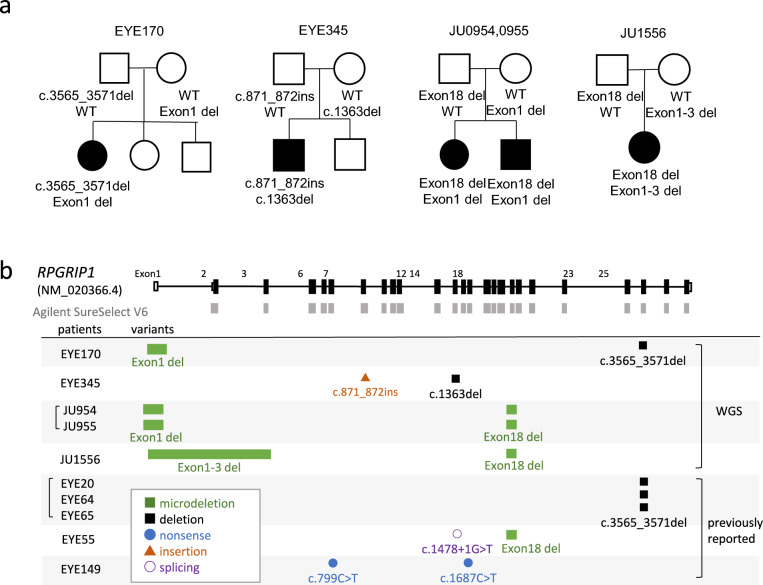
Pedigrees and schematic representation of *RPGRIP1* variants. **a** Pedigree of five patients with biallelic *RPGRIP1* variants. The segregation of each variant is shown. **b** Schematic representation of the *RPGRIP1* transcript (NM_020366.4) (b). The untranslated and coding regions are represented by open and filled rectangles, respectively. The baits of Agilent SureSelect V6 are shown in gray boxes. *RPGRIP1* variants of five patients diagnosed using whole-genome sequencing are shown together with those of five patients from our previous report [59]

We recently performed WES and whole-genome sequencing (WGS) on newly recruited cases with the above-mentioned criteria and unresolved cases from a previous study [59]. We identified LCA caused by *RPGRIP1* variants in five cases from four families. In particular, a 1339 bp deletion encompassing exon 18 of *RPGRIP1* was observed in three individuals from two families (Fig. 2). Consistent with a previous report, an exon 18 deletion in *RPGRIP1* was detected in six individuals from four families in Japan [56, 60]. An exon 18 deletion was also identified in a recently published database of Japanese cohorts. Because the allele frequency is relatively high at 0.002, this variant should be considered in genetic counseling for LCA/EOSRD. Although we analyzed a few patients, we believe *RPGRIP1* may be a common causative gene in Japanese patients with LCA/EOSRD.

Although making a simple comparison is difficult, a recent analysis of retinal dystrophies in a group of 411 family members under 18 years of age in the United Kingdom showed a prominent aberration of the *ABCA4* gene [61]. However, in our cohort, *ABCA4* gene abnormalities were found in only two families. These reports suggest that the frequency of the causative genes varies according to a patient’s ethnic background. Because of the limited number of cases, conducting additional research is imperative.

## Complex genes

### (1) Blue cone monochromacy (BCM)

**Fig 3.**
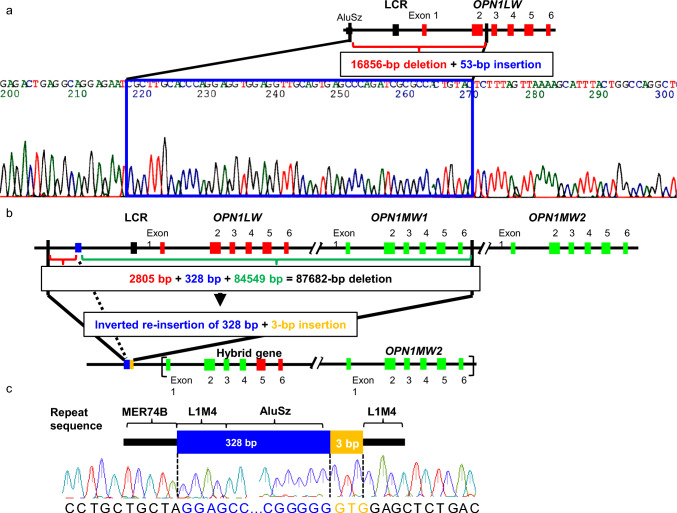
Determination deletion breakpoints in two Japanese families with blue cone monochromacy (BCM). **a** Analysis of the deletion–insertion mutation by junction PCR and sequencing in Case BCM1 (W family). Genomic structure of human *OPN1LW*. The red and black boxes represent the *OPN1LW* exons and locus control region (LCR), respectively. Case BCM1 has a 16,856-bp deletion and 53-bp insertion. The proximal and distal boundaries of the deletion are 8899 bp upstream of the *OPN1LW* translational start codon and within *OPN1LW* intron 2, respectively. The proximal boundary of the breakpoint is an *AluSz* repeat sequence; the 53-bp insertion contains a partial *Alu* repeat sequence. **b** Analysis of the deletion–inversion–insertion mutation by genomic walking in Case BCM2 (N family). Genomic structure of human *OPN1LW* and *OPN1MW* genes. The red and green boxes represent the *OPN1LW* and *OPN1MW* exons, respectively; the black box represents the LCR. Case BCM2 has an 87,682-bp deletion, an inverted 328-bp re-insertion that is a part of the deleted sequence, and a 3-bp insertion. The proximal and distal boundaries of the deletion are 28,144 bp upstream of the *OPN1LW* start codon and 7764 bp downstream of the *OPN1MW1* translation stop codon, respectively. In Case BCM2, exons 2 and 4 of *OPN1LW* are absent, whereas the presence of exon 5 in *OPN1LW* and *OPN1MW2* are confirmed. Case BCM2 may harbor a normal *OPN1MW2* gene and a hybrid gene comprising *OPN1MW2* exons 1–4 joined to *OPN1LW* exons 5 and 6. The brackets indicate that this structure is possible, including the order of the hybrid gene and *OPN1MW2*. **c** Diagram and electropherogram of the region surrounding the deletion–inversion–insertion breakpoints in Case BCM2, located within highly repetitive sequences [63]

Blue cone monochromacy (BCM, OMIM 303700) is a rare, X-linked genetic disorder associated with severe visual impairment. The *OPN1LW/OPN1MW* cluster on the long arm of the X chromosome (Xq28) encodes long-wave–sensitive and medium-wave–sensitive opsins. BCM is caused by an abnormality in the *OPN1LW/OPN1MW* gene cluster. We performed molecular genetic analysis of two families examined at Nagoya University (families N and W) [62, 63]. The *OPN1LW/OPN1MW* cluster consists of one *OPN1LW* gene and several *OPN1MW* genes in tandem. Since the sequences of the *OPN1LW* gene and the *OPN1LW* base sequence are almost identical, the analysis of the *OPN1LW* gene requires a certain level of ingenuity. The deletion in the W family shown in Fig 3a was relatively easy to identify, attributed to its extension from the promoter to the *OPN1LW* gene; the presence of a repetitive DNA sequence (*AluSz*) upstream of the deletion breakpoint was confirmed. In the N family shown in Fig 3b, a long region including at least the entire exon of the *OPN1LW* gene was deleted from 2.8 kb upstream of the gene to a breakpoint around 7.7 kb downstream of the gene. Between the two deletions, 328 bp of the deleted sequence was inverted, and three additional unknown bases were inserted. In addition to the deletions, partial duplication or non-homologous recombination occurred between *OPN1LW* and *OPN1MW1*. *OPN1MW2* may have caused the formation of hybrid genes; the exact genome structure could not be determined. Figure 3b shows an example of the putative genome structure of the N family. In common with the W and N families, the upstream, downstream, and inverted sequences of the deletion breakpoints contained high-frequency DNA repeats.

BCM is characterized by abnormalities in the *OPN1LW/OPN1MW* cluster and has a highly intricate cause, as indicated by the following findings: (1) major deletions and structural variations in the same gene cluster, (2) point mutations causing single or multiple loss of function in the same gene cluster, and (3) rare exon 3 haplotypes that cause splicing abnormalities in single or multiple copies of the gene [63–68]. The structure of the gene cluster complicates genetic diagnosis by PCR or next-generation sequencing. An ERG is more clinically useful than a genetic diagnosis since it can be diagnosed by ERG (RETeval^TM^), which has recently become available [69].

### (2) Incontinentia pigmenti with retinopathy

**Fig 4.**
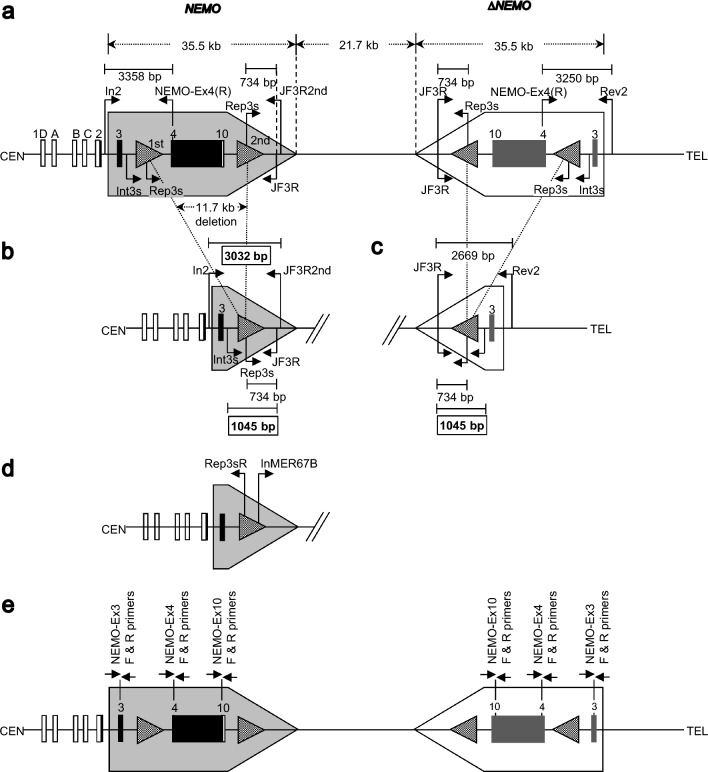
Genomic organization of *NEMO* vs. *ΔNEMO* and their recurrent deletion. **a** Tail-to-tail alignment of *NEMO* and *ΔNEMO* (a). In the *NEMO* gene, exons are shown as 1D, A–C, and 2–10; 1D and A–C indicate 4 alternative versions of exon 1: 1D (GenBank: NM_001377312.1), 1A (NM_001099856.6), 1B (NM_001321396.3), and 1C (NM_003639.4). Noncoding and coding exons are shown as white and black boxes, respectively. The longer black box of exons 4–10 also contains introns. On the *ΔNEMO* side, corresponding positions to each of the *NEMO* exons are shown except for the 4 alternative versions of exon 1, which are missing in *ΔNEMO*. The pentagons on the *NEMO* and *ΔNEMO* sides show the highly homologous duplicated sequences. The arrows with dotted lines indicate the lengths of the duplicated sequences and the distance between them. The identical MER67B repeats are shown as dotted triangular areas on both the *NEMO* and *ΔNEMO* sides. The PCR primers are shown by arrows with solid lines. The expected sizes of the PCR products are indicated by the solid lines with short bars at the ends. **b** The genomic structure of a *NEMO* allele generated by recurrent exon 4–10 deletion. An 11.7-kb sequence is lost due to the deletion. The PCR product size, specific to the recurrent exon 4–10 deletion, is indicated by an enclosing line (3032 and 1045 bp). **c** The genomic structure of a *ΔNEMO* allele generated at the position homologous to *NEMO*. **d** The sequence of the primers used to examine the genomic structure of the *NEMO* allele with the recurrent deletion. **e** The primer positions for the real-time PCR used to quantify the *NEMO* and *ΔNEMO* copy number. See reference [73] for the primer sequences

Incontinentia pigmenti (OMIM 308300) is an infantile disorder having rashes and ocular and central nervous system manifestations, with retinopathy in approximately 35% of cases [70]. It affects mainly females; male patients are rare. It is caused by a variant of the *IKBKG/NEMO* gene. With an X-linked dominant mode of inheritance, deletions of exons 4–10 in the *IKBKG/NEMO* gene are found in approximately 80% of affected individuals [71]. Male patients are reported to have somatic cell mosaicism [72]. Molecular genetic analyses of patients with retinopathy at the NCCHD, whose diagnoses were confirmed by skin biopsy, were conducted in cooperation with the Preeminent Medical Photonics Education & Research Center (Table 2). The causative gene for incontinentia pigmenti, *IKBKG/NEMO*, has a complex structure and includes pseudogenes (Fig. 4). Four patients, including a boy, showed a deletion of exons 4–10 in the known *IKBKG/NEMO* gene; subsequently, we identified a truncation of the deletion [73]. The deletion of P295 was de novo; the deletion of P356 was of maternal origin. Extensive sequencing of the exons and surrounding regions of P280 did not reveal any causative variants. No deletion variants were detected in the blood DNA of the male patients, but saliva DNA showed a mosaic pattern with a mixture of both deleted and undeleted *IKBKG/NEMO* genes (in approximately equal amounts in the samples collected at the time of the study). No deletion variants were detected in vital tissues, suggesting that *IKBKG/NEMO* is normal and explaining how the male patient is alive. More cases need to be documented to examine changes over time.

**Table 2:** Molecular genetic analysis of incontinentia pigmenti with retinopathy

Patient	Gender	Age	Family history	Ocular findings	Systemic findings other than skin	*IKBKG/NEMO* gene variants	Remarks
P262	Male	4 months	None	Retinopathy (OU)	malformed teeth	Exon 4–10 deletion	A mosaicism*
P280	Female	11 months	Mother with retinopathy	Retinopathy (OU)	malformed teeth	Not detected	
P295	Female	5 years	None	Retinopathy (OU)	malformed teeth	Exon 4-10 delation(de novo)	
P301	Female	5 years	None	Retinopathy(OS)	None	Exon 4-10 delation	
P356	Female	3 months	Mother with retinopathy	Retinopathy(OS), Iris cyst(OU)	None	Exon 4-10 deletion(maternal origin)	

All patients were confirmed by skin biopsy

^*^The boy’s saliva DNA showed a mosaicism consisting of the deletion and intact alleles, but his blood DNA did not. Relative quantification analysis of the real-time PCR data estimated the mosaicism ratio of the boy’s saliva as 45:55 (deletion:intact)

## Rare genotypes and rare phenotype

### (1) Isodisomy

Isodisomy is a rare genetic abnormality in which a chromosome from the father or mother is duplicated. Its cause is a biological reaction that prevents trisomy [74–76]. IRD due to isodisomy is rare; however, a few cases have been reported [77, 78]. Through a detailed review of genomic data, we identified two cases, a girl (Case 1) and a man (Case 2) [78].

**Fig 5.**
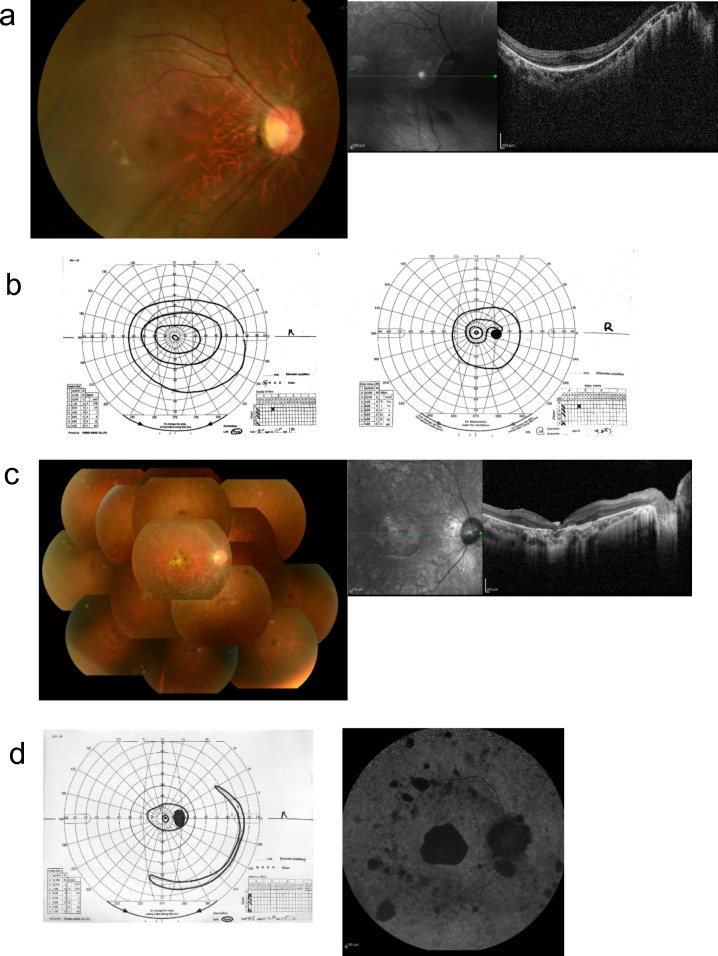
**a** Right fundus photography at 9 years of age (left) and an optical coherence tomography (OCT) image at 10 years of age (right). The fundus photography shows poor retinal color and narrowing of the retinal blood vessels. Although the OCT images are poor due to nystagmus, retinal thinning and the ellipsoid zone (EZ) line disappearance are observed. **b** The visual field is examined at 9 (left) and 14 years of age (right). The visual field is progressively constricted. **c** Right fundus photograph at 26 years of age (left) showing poor retinal color and narrowing of the retinal blood vessels and (right) an OCT image at 31 years of age showing the retina is highly thinning; the EZ line is not observable. **d** The visual field is examined at 26 years of age (left) and fundus autofluorescence (FAF) at 14 years of age (right). The visual field is severely constricted; the FAF shows low fluorescence, consistent with retinal degeneration [78]

Case 1 had nystagmus since birth and made a first visit to our hospital at 8 years of age. Fundus examination revealed narrowing of the retinal vessels and changes in the retinal pigment epithelium. Optical coherence tomography (OCT) showed severe thinning of the retina (Fig. 5). The visual field showed progressive afferent visual field constriction, and ERG had an obliterating pattern, leading to the diagnosis of early-onset retinal dystrophy. Genetic analysis suggested a maternal UPD on chromosome 4. With loss of heterozygosity, the patient showed a homozygous variant of the *SRD5A3* gene. The variant was in the transmembrane domain and substituted a conserved amino acid among vertebrates. We examined glucosylation using serum transferrin at the Osaka Women’s and Children’s Hospital, diagnosing the first congenital dysglucosylation type 1q (OMIM 612379) in Japan.

A 31-year-old male patient presented with photophobia and night blindness at 7 years of age and was diagnosed with RP at 10 years of age. The visual field showed severe centripetal constriction; fundus examination revealed narrowing of retinal vessels, numerous pigment spots in the middle periphery, and degeneration of the macular area (Fig. 5c, d). ERG showed an obliterating pattern. OCT showed severe retinal thinning; fundus autofluorescence (FAF) showed hypofluorescence, leading to the diagnosis of RP with early macular involvement. Genetic analysis suggested a maternal UPD on chromosome 8. Loss of heterozygosity was observed in chromosome 8, including the *RP1* gene; the Integrative Genomics Viewer showed a sharp decrease in sequencing depth in exon 4 of *RP1*, suggesting an *Alu* insertion. Based on these results, our diagnosis was *RP1-*related RP (OMIM 180100). This *Alu* insertion is a causative variant of the disease recently reported [50]. We consider it a major cause of *RP1-*related retinal dystrophy in Japan [49, 79]. A homozygous *Alu* insertion was confirmed in the patient by PCR; the mother was a carrier of the insertion. Most cases of IRD due to isodisomy occur in children. This finding should be considered during genetic counseling for IRD in children.

### (2) Juvenile-onset retinal dystrophy caused by double mutations

**Fig 6.**
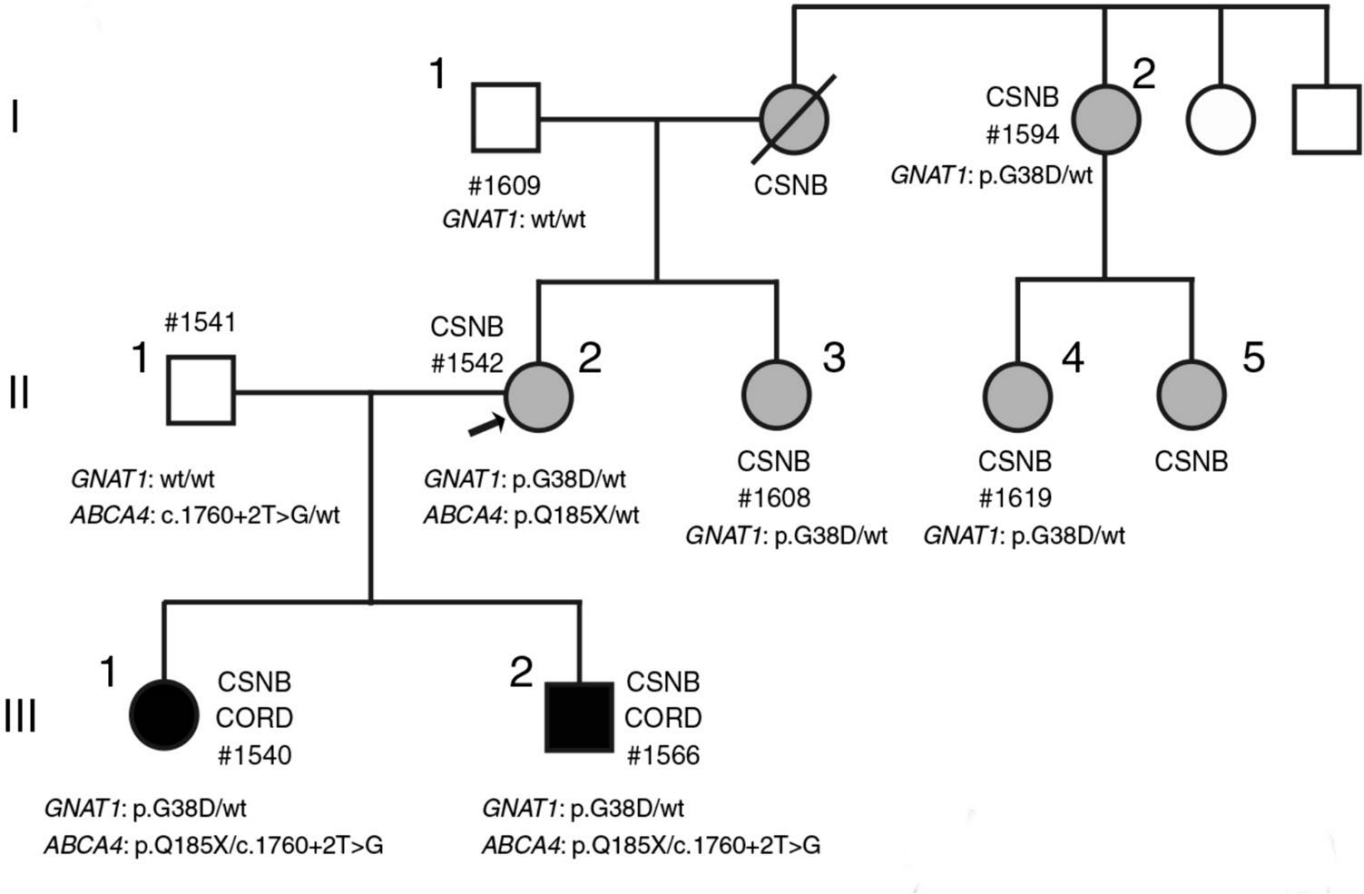
Pedigree of a Japanese family showing nucleotide sequences of exon 2 of the *GNAT1* gene. Unaffected members are shown as unfilled circles (female) or squares (males); patients with congenital stationary night blindness (CSNB) or cone-rod dystrophy (CORD) are shown as gray or black symbols, respectively. The female proband (II-2) is indicated by an arrow; ‘‘wt’’ denotes the wild-type genotype. A heterozygous *GNAT1* variant (p.G38D) is found in all examined patients with CSNB, including the proband. The patients with CORD (III-1) carry compound heterozygous *ABCA4* variants (p.Q185X and c.1760+2>G) [83].

**Fig 7.**
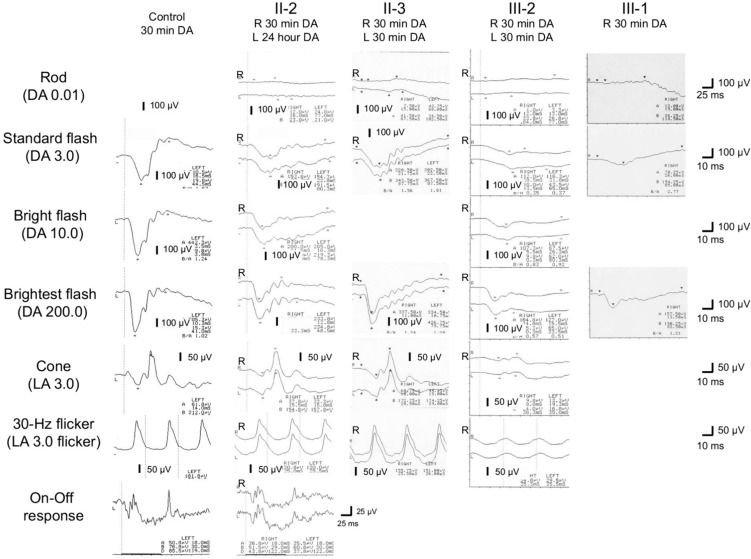
Full-field electroretinography (ERG) findings of a control participant, two patients (II-2 and II-3) with congenital stationary night blindness (CSNB), and two patients (III-1 and III-2) with cone-rod dystrophy (CORD). The ERG of patient II-2 (CSNB) shows a non-recordable rod response (dark-adapted 0.01 cd·s·m^-2^; DA 0.01) but at least 50% greater than normal a- and b-wave responses in DA 3.0, DA 10.0, and DA 200 ERG after 30 min of DA in the right eye and 24 h of DA in the left eye. The cone (light-adapted 3.0 cd·s·m^-2^; LA 3.0) and 30-Hz flicker (LA 3.0 flicker) responses are normal. Clear on and off responses are observed. The ERG findings of patient II-3 (CSNB) are comparable with those of patient II-2. In patients III-2 and III-1 (CORD), the DA ERG shows a non-recordable rod response (DA0.01) and severely decreased a- and b-wave responses to a strong flash (DA 3.0) but only about one-third of the normal response to a greater flash (DA 200). The LA 3.0 and LA 3.0 flicker responses are severely decreased in III-2, consistent with CORD features [83]

Double mutations have been documented in diverse medical conditions. Some reports come from the ophthalmology field [80–82]. The proband was a patient with congenital stationary night blindness (CSNB) at the Jikei University School of Medicine in Tokyo (Patient II-2 in Fig. 6); two children (Patients III-1 and III-2) showed abnormal retinal dystrophies in both cones and rods [83]. Patient III-1 showed a grayish color change within the vascular arcade; Patient III-2 showed no abnormality. FAF showed a ring of hyperfluorescence. OCT showed thinning of the retina in the macular area with loss of the ellipsoid zone (EZ) line. The ERGs of Patients II-2 and II-3 with the International Society for Clinical Electrophysiology of Vision protocol were generally consistent with the ERG of Nougaret-type CSNB (OMIM# 610444) (Fig. 7), including obliterated rod responses, normal cone responses, and clear ON-OFF responses [84, 85]. Patients III-2 and III-1, who had juvenile-onset cone-rod dystrophy, showed reduced cone and 30-Hz flicker responses in addition to the loss of rod responses. Genetic testing of the family history revealed a heterozygous p.G38D missense variant of the *GNAT1* gene, which may have caused the rod abnormality. Furthermore, we identified a compound heterozygous variant of spliced and nonsense mutations in the *ABCA4* gene in Patients III-1 and III-2 in addition to the missense *GNAT1* variant. We consider that Patients III-1 and III-2 have the phenotype of juvenile-onset cone-rod dystrophy overlapping with Nougaret-type CSNB.

### (3) LHON-like optic neuropathy in patients with RP due to COQ2 gene abnormality

**Fig 8.**
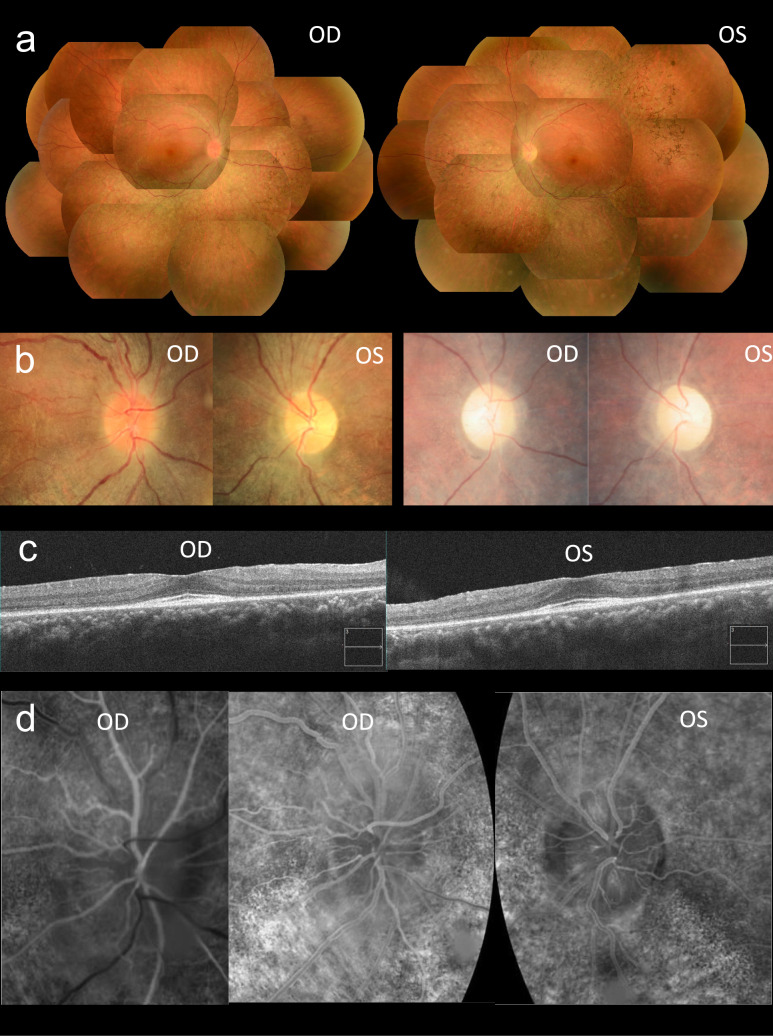
**a** Fundus findings of a patient. Color fundus photography at the first visit shows diffused retinal degeneration, including bone–spicule pigmentation and narrowed retinal vessels. **b** Optic disc findings of the patient. The optic disc is hyperemic in the right eye and pale in the left eye at the first visit (left row). The optic discs are pale in both eyes at the last visit (right row). **c** OCT findings of the patient. OCT at the first visit reveals relatively preserved inner retinal layers. Thinning of the outer nuclear layer and disruption of the EZ line are seen from the parafoveal area to the surrounding area. **d** Fluorescein angiography at the first visit reveals peripapillary telangiectatic blood vessels in the early phase in the right eye (left row) and neither staining nor leakage around the optic disc in the late phase in both eyes (middle and right row). OD: oculus dexter; OS: oculus sinister [88].

A 27-year-old male patient was referred to us for genetic counseling for RP. Before visiting our hospital, the patient expressed concerns about vision difficulties in his left eye. A diagnosis of RP was made based on visual field and fundus findings. Redness was observed in the right optic nerve papilla, and fluorescence fundus angiography showed telangiectasia around the optic nerve papillary vessels in the right eye (Fig. 8). The correlation between treatment and visual function was similar to that reported in 1993 for LHON, in which visual acuity was restored [4]. A case of double mutation in the *RP2* gene and the 11778 mutation of the mitochondrial gene had been reported [86]. We performed a genetic analysis of this case, suspecting it might be a similar abnormality. Although no variant was found in the mitochondrial gene, whole exome analysis revealed a known compound heterozygous missense mutation in the *COQ2* gene. RP due to mutations in the *COQ2* gene has been reported [87]. COQ2 is an enzyme essential for the biosynthesis of COQ10. We propose that the *COQ2* mutations cause mitochondrial electron transfer system dysfunction, resulting in LHON-like optic neuropathy [88].

An autosomal recessive form of LHON (LHONAR1, arLHON, MIM: #619382) has recently been reported. The autosomal recessive form of LHON is caused mainly by biallelic variants in the *DNAJC30* gene (MIM: 618202) [89, 90]. Typical LHON phenotypes have also been reported to be caused by variants in other genes: *NDUFS2*, *MCAT,* and *NDUFA12*, detected only in a few families [90]. The prevalence of LHON caused by these gene variants in Japanese patients is unknown. Pathogenic variants of the *DNAJC30* gene are reported to affect the mitochondrial complex I subunit and interact with mitochondrial adenosine triphosphate synthesis [89]. Mitochondrial electron transport chain dysfunction is significantly involved in LHON pathology.

*COQ2* gene variants are known to cause multiple system atrophy [87]. This phenomenon is called an incidental finding/secondary finding (IF/SF) and is a major issue to be considered when expanding the scope of genetic testing. No systemic abnormalities have been observed in the patient in which this was identified except for a relatively low platelet count; however, the patient is being carefully monitored by a neurologist.

Most inherited eye diseases are caused by one variant in the cases of dominant inheritance or two variants in the cases of recessive inheritance. Although rare, complex genetic abnormalities also occur. Multiple variants can affect the disease phenotype, or one variant may be involved in several diseases; defining the effect of each variant is presently difficult.

## Clinical application

### (1) Genetic counseling for genetic diseases in Japan

**Fig 9.**
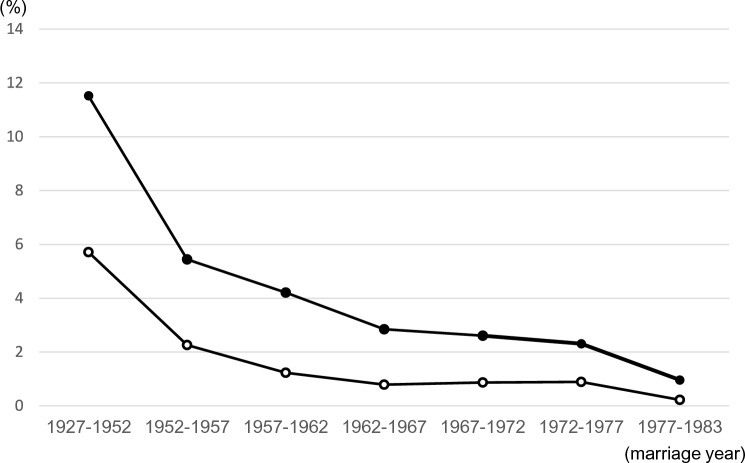
Rate of consanguineous marriage in Japan. ●: Rate of consanguineous marriage (%). ○: Rate of cousin marriage (%). Among advanced countries, Japan had an exceptionally high number of incestuous marriages, but the number has decreased rapidly in recent years [21, 91]

At the beginning of the 20th century, consanguineous marriages in Japan were much more common than in other industrialized countries, but they have rapidly decreased [21, 91] (Fig. 9). Avoiding consanguineous marriages is crucial, as is continuing educational efforts on this subject as a proactive measure in managing hereditary eye diseases. We collaborated with the Department of Otorhinolaryngology, Hamamatsu University School of Medicine, and the Preeminent Medical Photonics Education & Research Center for genetic analysis of Usher Syndrome [92–95]. The *USH2A* gene is a representative causative gene of Usher syndrome. We have already described the *USH2A* gene analysis of RP without hearing loss before. The results of the *USH2A* gene analysis of the otorhinolaryngology cohort were all compound heterozygous variants [92]. Although preventing hereditary eye diseases caused by compound heterozygous variants is difficult, risk assessment by genetic testing of spouses and genetic counseling are promising countermeasures.

### (2) Predicting the prognosis of inherited eye diseases

Clarifying the causative variant facilitates predicting the prognosis. Taking IRD as an example, three types of genetic abnormalities occur: (1) those causing significant visual impairment from infancy, (2) those causing problems in childhood, and (3) those causing problems in adulthood. Further documentation of cases and the clinical identification of each variant will enable prognoses to be predicted more accurately.

**Fig 10.**
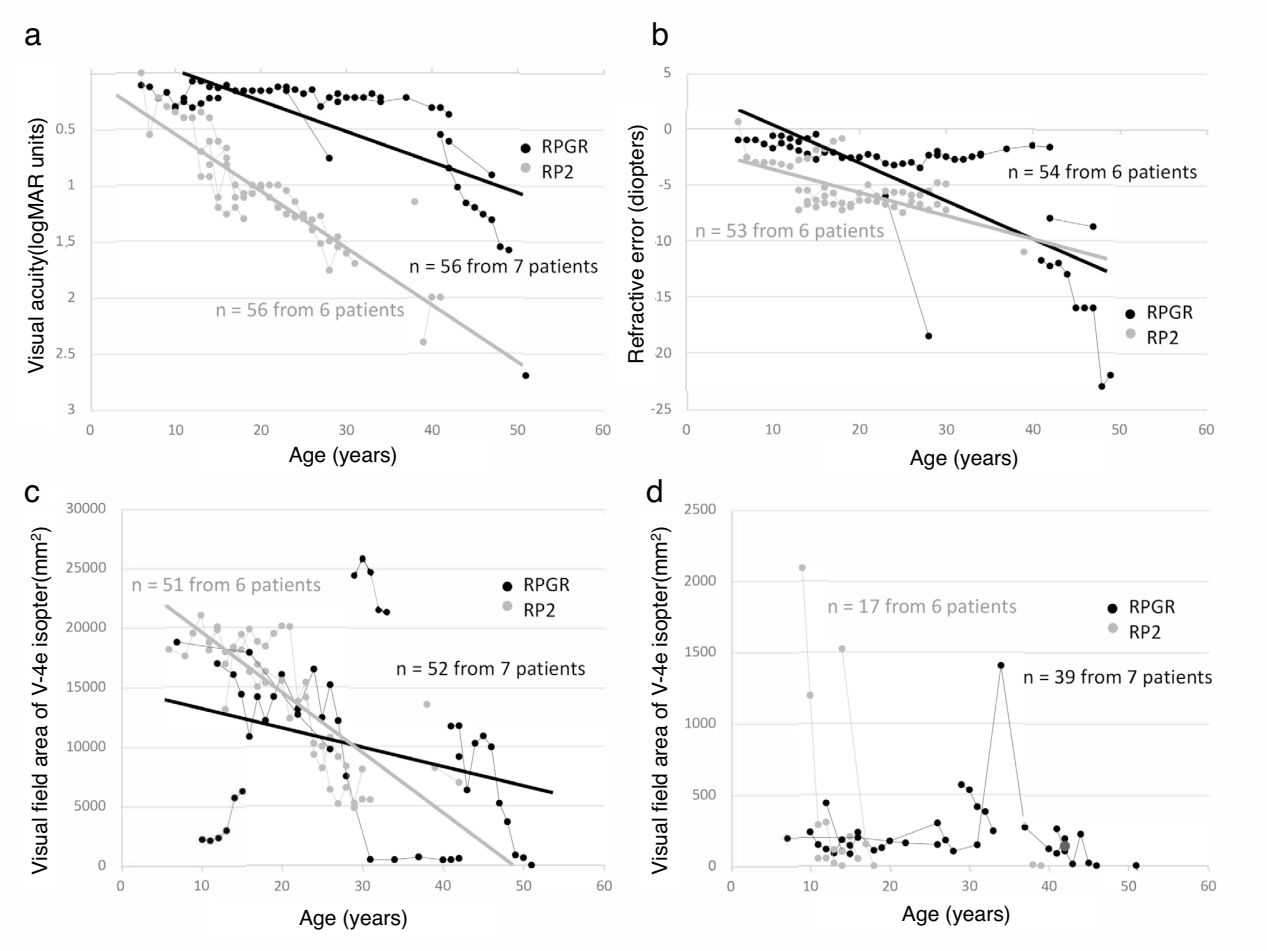
Correlation between visual function and age among affected Japanese patients with X-linked RP. **a** The graphs show visual acuity values expressed in logMAR units, **b** refractive error, **c** visual field extent with the V-4e isopter, **d** and visual field extent with the I-4e isopter versus age. The data from the same participant are indicated by bars connecting the points [97]

Family tree analysis identified that XLRP is rare in Japan [96]. However, genetic diagnoses revealed that variants of the *RPGR* and *RP2* genes were found in isolated cases; large-scale analyses revealed they are not rare [41–43]. We examined the clinical profile of patients from 12 families with causative *RPGR* (n=7) and *RP2* (n=5) variants and female carriers [97]. Male patients had severe visual dysfunction, and the *RP2* variant was slightly more severe (Fig. 10).

Female carriers of XLRP vary from being asymptomatic to severely impaired. FAF shows patchy radial hyperfluorescence, which is more common in wide-angle imaging [98, 99]. Attempts have been made to grade carriers of XLRP according to fundus findings. Table 3 shows the visual function of XLRP carriers by grade of fundus finding. The visual function of carriers of XLRP with severe myopia is considered more severe in older patients. Some patients diagnosed with severe myopic chorioretinal atrophy may be carriers of XLRP. The reason why carriers of XLRP with high myopia are affected more severely is unknown.

**Table 3.** Relation of fundus grade and visual function in female carriers of XLRP [97]

	Grade 0	Grade 1	Grade 2	Grade 3	J-T Test
Age (years)	53	35.7	62.6	67.6	p = 0.001 **
BCVA (logMAR unit)	− 0.015	− 0.079	0.2	0.47	p = 0.005 **
Refractive error (diopters)	− 1.81	− 3.29	− 5.06	− 14.04	p = 0.001 **
Visual field area (mm^2^)					
V-4e isopter	20619	22716	21540	12439	p = 0.046 *
I-4e isopter	9343	11443	10312	6198	p = 0.062

*J-T* Jonckheere–Terpstra; *: significant at p < 0.05, **: significant at p < 0.01

### (3) Gene therapy

Since the 1980s, gene transfer methods have been studied for hereditary eye diseases [100–102]. Clinical trials underway for IRD in Europe and the USA use mainly adeno-associated virus (AAV) vectors [103, 104]. Clinical trials for LCA caused by *RPE65* gene abnormalities have also started in Japan. Although developing a treatment effective for all types of IRD is desirable, most investigations are focused on treatment methods specific to the causative gene. AAV vectors are relatively safe because they exist as episomes and do not incorporate into the host’s DNA [105, 106]. However, in addition to long-term efficacy, developing drugs that are safe even when administered for a long period is desirable.

*EYS* gene abnormalities are among the most important causes of RP leading to blindness in Japan. The *EYS* gene-related patients can often adapt to society until 50 years of age. If the rate of progression can be halved, they may be able to adapt throughout their lives. The problem in developing a treatment for RP is that the *EYS* gene does not exist in most other mammals, making animal experiments difficult. In addition, the gene is so large that inserting it into a vector is difficult. We have observed and reported retinal dystrophies over a long period [107–109]. Understanding the natural course of the disease is imperative for accurately assessing the efficacy of treatment interventions.

## Conclusion

Ocular genome research has progressed rapidly over the past 30 years. The knowledge of ocular genetics has become vast and continues to grow. Genetic counseling, prognosis prediction, and gene therapy have shown effectiveness in patient interventions. Although expectations are high, many issues need to be considered, including the social and familial stigma of a genetic diagnosis, IF/SF, long-term effects of gene therapy, and safety. By understanding patient diversity through genetic diagnoses and formulating parameters to assess responses to both the environment and drugs, the efficacy of treatments for hereditary eye diseases can be rapidly determined. This approach enables establishing evidence-based methods for addressing these conditions within a limited timeframe. Expectations for the future of precision medicine extend beyond the scope of genetic diagnoses. Above all, providing patients suffering from intractable diseases with hope and accurate information is crucial.
